# Practical implications for the quality assurance of modulated radiation therapy techniques using point detector arrays

**DOI:** 10.1002/acm2.12157

**Published:** 2017-08-30

**Authors:** Steffi Kantz, Almut Troeller McDermott, Matthias Söhn, Sabine Reinhardt, Claus Belka, Katia Parodi, Michael Reiner

**Affiliations:** ^1^ Department of Radiation Oncology University Hospital LMU Munich Munich Germany; ^2^ Department of Radiation Oncology Beaumont Health Royal Oak MI USA; ^3^ Faculty of Physics Department of Medical Physics LMU Munich Munich Germany

**Keywords:** 2D arrays, IMRT QA, linac‐oriented QA, VMAT QA

## Abstract

**Purpose:**

Linac parameters potentially influencing the delivery quality of IMRT and VMAT plans are investigated with respect to threshold ranges, consequently to be considered in a linac based quality assurance procedure. Three commercially available 2D arrays are used to further investigate the influence of the measurement device.

**Methods:**

Using three commercially available 2D arrays (Mx: MatriXX^evolution^, Oc: Octavius^1500^, Mc: MapCHECK2), simple static measurements, measurements for MLC characterization and dynamic interplay of gantry movement, MLC movement and variable dose rate were performed. The results were evaluated with respect to each single array as well as among each other.

**Results:**

Simple static measurements showed different array responses to dose, dose rate and profile homogeneity and revealed instabilities in dose delivery and profile shape during linac ramp up. Using the sweeping gap test, all arrays were able to detect small leaf misalignments down to ±0.1 mm, but this test also demonstrated up to 15% dose deviation due to profile instabilities and fast accelerating leaves during linac ramp up. Tests including gantry rotation showed different stability of gantry mounts for each array. Including gantry movement and dose rate variability, differences compared to static delivery were smaller compared to dose differences when simultaneously controling interplay of gantry movement, leaf movement and dose rate variability.

**Conclusion:**

Linac based QA is feasible with the tested commercially available 2D arrays. Limitations of each array and the linac ramp up characteristics should be carefully considered during individual plan generation and regularly checked in linac QA. Especially the dose and dose profile during linac ramp up should be checked regularly, as well as MLC positioning accuracy using a sweeping gap test. Additionally, dynamic interplay tests including various gantry rotation speeds and angles, various leaf speeds and various dose rates should be included.

## 1 INTRODUCTION

Implementing modulated radiation therapy techniques like step‐and‐shoot intensity modulated radiation therapy (sIMRT), dynamic sliding window IMRT (dMLC), or volumetric modulated arc therapy (VMAT) into clinical routine requires generating a correct beam model of the linac in the treatment planning system (TPS) used. It also requires verification of this beam model and effective quality assurance (QA) methods in order to keep the linac in accordance with the beam model and to ensure that TPS generated treatment plans are correctly calculated by the beam model.

For beam modeling careful measurements of quadratic and rectangular open fields with a single detector in a large water tank and some simple tests for MLC parameter definition are required. For these simple tests, point detector arrays or electronic portal imaging devices (EPIDs) are frequently used. The beam model is built according to these measurements and subsequently verified by measuring a sequence of simple to complex plans. Often the same measurement devices that were used for the data collection are used. After establishing the beam model, the regular plan‐individual and linac QA measurements are also conducted with these devices.

During the last years, measurement devices have largely improved with regard to resolution 1, 2, 3 and needs for rotational techniques like VMAT.^4^ In addition, improvements in tests for the correct beam model parameters were proposed.^5^, ^6^, ^7^, ^8^ Nevertheless, the evaluation of treatment plan QA is still cumbersome as shown by ongoing discussions about the widely used gamma‐evaluation method itself, the optimal evaluation parameters and the comparison between different measurement devices.^9^, ^10^, ^11^, ^12^, ^13^, ^14^, ^15^, ^16^, ^17^, ^18^

Bypassing the measurement, linac log files, which monitor each single parameter of the linac, can be used to reconstruct the delivered dose.^19^, ^20^, ^21^ As linac log files monitor surrogate parameters for absolute linac values, miscalibrations could stay undetected without tight linac QA. Furthermore, the sampling rate might be too low in some cases to reveal errors in fast changing parameters like leaf acceleration, which was shown to have a large impact on good agreement between calculation and delivery.^22^, ^23^

Besides plan‐individual measurements and evaluation of linac‐log files, delivery subsystems such as MLC movement or dose rate stability can be analyzed separately in a linac based QA approach. This means that the complexity of especially VMAT is split into its single significant components and that measurements are conducted without devices that are developed especially for the rotational needs in VMAT. Instead, known devices (e.g. 2D arrays) are used. During commissioning, this simplification points out general linac parameters that are potentially subject to influence the delivery quality of IMRT and VMAT and thus should be respected throughout the treatment plan generation. As a consequence, delivery mismatch with respect to the TPS calculation can be modeled and quantified, thereby establishing action levels. With 2D array measurements, we show how the splitting of complexity into significant parameters, that may influence IMRT and VMAT delivery quality, enables effective and efficient regular QA of delivery subsystems. This can provide information about the actual linac conditions, which can be used to ensure good treatment plan delivery.

Thus, we propose an efficient, fast and meaningful linac based QA approach without extensive, cumbersome and time‐consuming plan individual QA by decomposing the complexity of IMRT and VMAT and evaluating the delivery performance of the linac. As different linacs may behave differently and guidelines may require department specific limits, our work focuses on the workflow of identifying linac parameters limiting the delivery quality and separating them from restrictions of measurement devices. Three different 2D arrays are used as an example for how to separate restrictions of the measurement device from the limitations of the linac delivery, which can be translated to other measurement devices. Eventually, the limiting parameters and their safe ranges need to be carefully considered during beam modeling and could be implemented to the TPS in order to guarantee IMRT and VMAT plans with safe delivery parameters.

## 2 MATERIAL AND METHODS

### 2.A Complexity levels in IMRT and VMAT QA

We break down the complexity of modulated radiation therapy techniques into three categories to investigate threshold ranges that may potentially limit plan delivery quality. These comprise linac parameters which should be included into plan generation as well as measurement shortcomings using 2D arrays:

1. Simple static QA tests (Table 1A): Treatment planning systems assume linearity of dose and dose rate as well as independence of the dose profiles from the delivered dose and dose rate. Furthermore, dose output with respect to the field size (output factors) is measured once and assumed constant over time. With simple static tests, these assumptions are verified with respect to the delivery accuracy of the linac as well as with respect to each array's capability of measuring these assumptions. As IMRT and VMAT plans may introduce several (differently shaped and steep) dose gradient areas, the resolution capability of the different arrays is verified by measuring the penumbra of a static field, investigating each array's potential of correct dose measurements. Therefore, these simple static tests may reveal general threshold ranges for each tested parameter, which may degrade the delivery and measurement quality of IMRT and VMAT plans.
  **Table 1:** Three level QA: simple static QA tests (A) serve as inspection of the measurement limits of the used three detector arrays. MLC parameter measurements (B) explore the different assumptions for TPS modeling. Dynamic interplay measurements (C) ensure the correct dose delivery at gantry speed, leaf speed and dose rate limits

Test	Field size [cm × cm]	MU	Dose rate [MU/min]	Evaluation
A) simple static QA tests				
Dose profiles Dose linearity Dose rate linearity	25 × 25	2 → 100	25 → max	homogeneity of in‐/crossplane profiles dose@iso rel. to 100 MU dose@iso rel. to DR_max_
Penumbra	10 × 10	100	max	penumbra, field size
Output factors	1 × 1 → 30 × 30	100	max	dose@iso rel. to 10 × 10^a^
Small dose summation	25 × 25	8 × 2, 4 × 4, 2 × 8	max	dose@iso rel. 1 × 16 MU
B) MLC parameters				
Static picket fence	12 adjacent segments: 2^b^ × 30	30/segment	max	crossplane profiles
Sweeping gap	2^b^ × 30 swept over 20 cm	100, 500, 1000	max	dose rel. to open field: 20 × 20, 100 MU
“chair” test 6		54	max	3 in‐ and 3 crossplane profiles
C) dynamic interplay				
Arc length (rot. velocity)	Leaf movement [cm] (leaf velocity)	MU	Dose rate [MU/min]	Evaluation
C1) leaf movement and dose rate				
0	2 × 30 swept over 20 cm	5, 10 →100^c^	50→max	dose rel. to open field: 20 × 20, 100 MU, crossplane profiles
C2) gantry movement and dose rate				
Q1: 20→70 (max)	0 (static open field: 20 cm × 20 cm)	9	min	dose rel. to same field w/o gantry rotation, crossplane profiles
Q2: 290→340 (max)		75	max	
Q3: 250→200 (min)		50	min	
Q4: 160→110 (min)		460	max	
C3) gantry and leaf movement with dose rate				
A: 178→160 (max)	18 (max)	3	min	dose rel. to same sweeping gap w/o gantry rotation, crossplane profiles
B: 180→180 (max)	6 (min)	60	min	
C: 182→200 (max)	18 (max)	27,5	max	
D: 180→180 (max)	6 (min)	550	max	
E: 90→86 (min)	24 (max)	4	min	
F: 330→30 (min)	6 (min)	60	min	
G: 275→278 (min)	18 (max)	27,5	max	
H: 60→0 (min)	6 (min)	550	max	

dose@iso: mean of the innermost 4 (Mx) or 5 (Oc, Mc) detectors of the array.

a for field sizes ≤5 cm × 5 cm, only one detector was used; for Mx this was achieved by shifting the array such that one ion chamber was at the isocenter.

b leaf manipulations: 0.1→1.0 mm in steps of 0.1 mm.

c in steps of 10 MU.
2. MLC parameters (Table 1B): MLC positioning is one of the most crucial concerns in radiation therapy, especially in modulated techniques, and a reliable test using either measurement method is required for regular MLC QA. As either the picket fence or the sweeping gap test will be used for this regular QA, we explore the power of identifying leaf mispositioning in the range of ±0.1–1.0 mm (in steps of 0.1 mm) with these two tests using three different arrays. Besides positioning, the beam model requires the correct differentiation between leaf transmission, leaf tip effects, and leaf position calibration, which is especially important for dynamic modulated techniques like dMLC and VMAT. Van Esch et al.^6^ proposed an efficient “chair” test that we adapted to work with Elekta Agility MLCs.
3. dMLC plans include different combinations of dose rate and leaf movement, VMAT plans include different combinations of dose rate and leaf movement plus gantry movement. A meaningful QA approach should guarantee that for all possible combinations the delivered dose remains stable over time. Therefore, we introduced tests, which check consistent dose delivery with different combinations of these parameters at their (supposed) minimal and maximal range by dynamic interplay tests (Table 1C). The maximal leaf speed is set at the manufacturer's specification to 6.0 cm/s. The minimal speed is assumed to be 0.1 cm/s after having investigated several clinical VMAT plans. The maximal gantry rotation speed is 6°/s by regulation. A reasonable minimal gantry rotation speed is assumed to be at 1°/s for these studies. The maximal dose rate of the used linac was around 550 MU/min and the minimal dose rate with stable dose profiles was found around 60 MU/min. To verify all combinations three subcategories are introduced, which concern the dynamic interplay of leaf movement and dose rate (C1), gantry movement and dose rate without MLC movement (C2) as well as with slow and maximal MLC speed (C3). Consistent dose delivery is checked at gantry angle 0° with maximal dose rate with respect to the particular open field, if no leaf or gantry movement was involved, or to the particular sweeping gap field, if leaf and gantry movement was involved.

### 2.B Measurement devices and setup

Similar consistent measurements for the three commercially available 2D arrays MatriXX^evolution^ (Mx, iba dosimetry, Schwarzenbruck, Germany), Octavius^1500^ (Oc, PTW, Freiburg, Germany) and MapCHECK2 (Mc, SunNuclear, Melbourne, FL, USA) are conducted. The main characteristics of these arrays are shown in Table 2.

**Table 2:** Characteristics of the used 2D arrays

Array	MatriXX^evolution^	Octavius^1500^	MapCHECK2
Detector type	Ion chamber		N‐type Si diode
Array size	24.4 × 24.4 cm²	27 × 27 cm²	32 × 26 cm²
Number of detectors	1020	1450	1527
Detector size	4.5 mm(d) × 5.0 mm³ = 0.08 cm³	4.4 × 4.4 × 3.0 mm³ = 0.06 cm³	0.8 × 0.8 mm² = 0.64 mm²
Arrangement	Cartesian	‘double Cartesian chess board’ shifted by 5 mm horizontal and vertical	
Distance between detectors	7.62 mm	7.07 mm (diagonal) 10 mm (horizontal and vertical)	
Eff. depth of measurement	0.3 cm	0.8 cm	2.0 cm

All measurements were carried out using a 6 MV photon beam of an Elekta Synergy linac equipped with an Agility^TM^ multi‐leaf‐collimator (MLC, leaf width: 5 mm). Before each measurement session, the arrays were dose calibrated at 5 cm depth.

For the simple basic QA tests (A) and the leaf‐dose‐rate‐interplay measurements (C1) the arrays were setup isocentric (source‐to‐detector‐distance SDD = 100 cm), with 10 cm additional backscatter material (RW3, PTW) and the measurement depth was raised to 5 cm (water equivalent) for all arrays using RW3. For the penumbra measurements, the arrays were shifted mm‐wise 10 times in in‐ or crossplane direction using a robotic table top.

**Figure 1:**
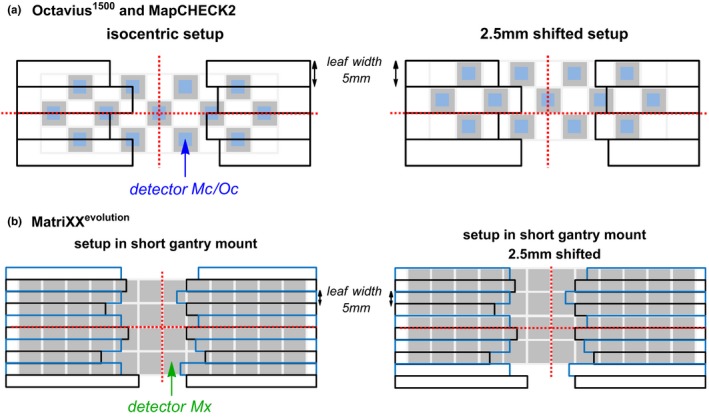
Schematic overview of the measurement setup of the different arrays (a) Oc and Mc, (b) Mx. Shifting the arrays accordingly enables the examination of single leaf pairs using a picket fence or sweeping gap test. When using Mx in the short gantry mount (SSD = 76.2 cm) each detector projects to 1 cm in the isocenter plane.

For the MLC parameter measurements (B) the arrays Oc and Mc were shifted half a leaf width (2.5 mm) in inplane direction to align each detector row exactly under one leaf [Fig. 1(a)]. The array Mx was setup in a short gantry mount (SDD = 76.2 cm) for these measurements such that the detector dimensions correspond to 1 cm in the isocenter and one detector row aligns with two leaves [Fig. 1(b)]. Additionally, the leaves were altered (±0.1→1.0 mm in steps of 0.1 mm) for the static picket fence and the sweeping gap test (field with 100 MU), to evaluate each array's ability of leaf misalignment detection.

For all measurements including gantry rotation (C2 + 3), the arrays were setup in either the corresponding gantry mount (Mx: SSD = 76.2 cm, Oc and Mc: SSD = 100 cm) or in a cylindrical phantom (Oc4D: Octavius4D phantom).

## 3 RESULTS

### 3.A Simple static QA tests

#### 3.A.1 Dose linearity

**Figure 2:**
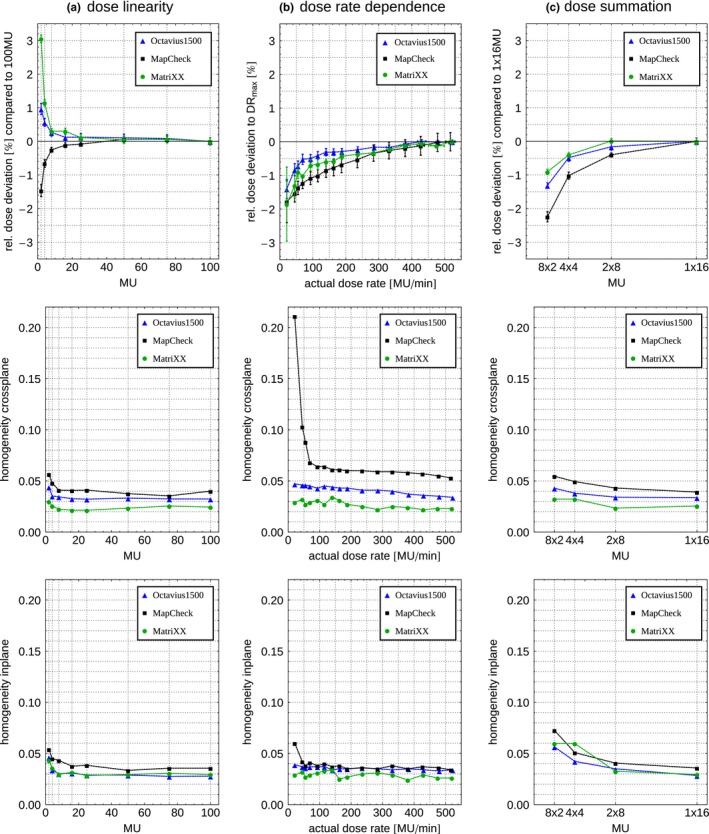
Relative deviation (as defined in Table 1), homogeneity of cross‐ and inplane profiles for (a) dose linearity, (b) dose rate dependence, and (c) dose summation using Mx (green), Oc (blue) and Mc (black).

For doses ≥8 MU (=8 cGy in this measurement setup), the deviation from the measured dose for 100 MU was ≤0.5% for all arrays [Fig. 2(a)]. For smaller doses the deviation for Oc was not larger than 1.0%, which is in accordance with measurements using a Farmer‐type ion chamber. For Mx and Mc, the deviation increased up to 3.1% and −1.5% respectively. While the deviation for small doses was always positive for Mx and Oc, Mc measured relatively less dose compared to the 100 MU measurement.

#### 3.A.2 Dose rate dependency

Compared to dose measured with the maximal dose rate, all three arrays measured lower dose for lower dose rates [Fig. 2(b)]. Using a Farmer‐type ion chamber, the deviation is not larger than −0.2%. For Mc, the deviation was largest within the three arrays. This deviation was not larger than 1% for dose rates larger than 75 MU/min and up to 1.9% for the smallest dose rate tested. Oc showed the lowest deviation with up to 1.4% for the smallest dose rate and less than 1% for dose rates larger than 50 MU/min. If the dose rate was not calibrated at the maximal possible dose rate but at 200–300 MU/min, the dose deviation for larger dose rates would be positive but would also not exceed 0.5%–1% for all arrays. On the other hand the dose deviation for smaller dose rates would also stay within −1% for all arrays. With regard to actually delivered dose rate levels in modulated techniques, this might contribute to less influence of the dose rate linearity of the arrays to the delivery quality of IMRT and VMAT plans.

#### 3.A.3 Profiles

While all arrays measured almost the same homogeneity in inplane direction, Oc and Mc measured more distinct shoulders on the left side, resulting in less homogeneity as Mx (Fig. 2 – middle and bottom panel). Furthermore, it was found that the measured homogeneity of the profiles decreases for low doses (≤8 MU) for all arrays as well as for low dose rates (≤60 MU/min), especially when using Mc. This effect is more pronounced for the crossplane profiles. Also for less influence of the profile homogeneity to the measurements, a calibration of the dose profile around the most often used dose rate could improve the overall delivery quality. Remarkable is that for Oc, not exactly flat RW3 slabs can disturb the measurement. Due to a minimal convex shape, the pressure on ion chambers in the middle of the arrays reduces the active volume and therefore the measured dose. This can be avoided by either flipping the convex slab 180° or by introducing a minimal space of about 1 mm between the array and the slabs.

#### 3.A.4 Summation of small doses

All summed doses were smaller than the dose measured for 1 × 16 MU [Fig. 2(c)]. The deviation relative to 1 × 16 MU was largest for Mc, but not larger than −0.9%, −1.3%, and −2.2% for Mx, Oc, and Mc respectively.

#### 3.A.5 Measurement of output factors

For field sizes ≥2 × 2 cm² no difference between the arrays and the according base data measurements (Farmer‐type ion chamber for field sizes larger than 5 × 5 cm², pinpoint detector for smaller field sizes) was found. Due to the volume averaging of the ion chambers, Mx and Oc measure 76% and 13% less dose compared to Mc and the according base data measurement for 1 × 1 cm².

#### 3.A.6 Dose gradient measurement

**Figure 3:**
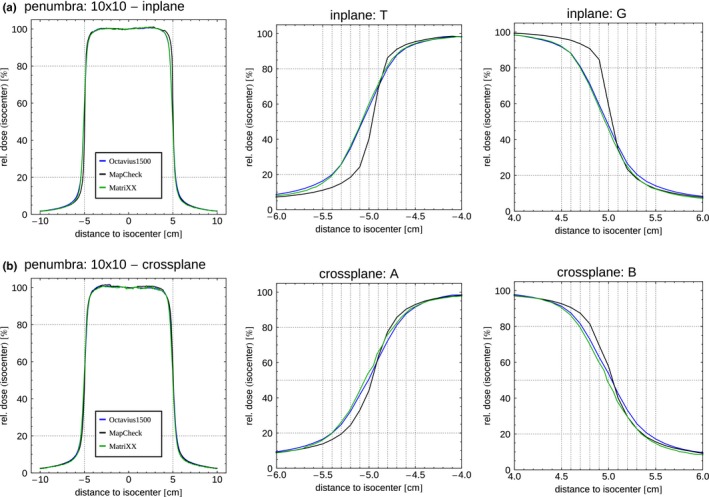
Measured penumbra resulting from shifting Mx (green), Oc (blue) and Mc (black) mm‐wise in a) in‐ and b) crossplane direction.

As a surrogate for dose gradients, the penumbra of a 10 cm × 10 cm field was used (Fig. 3). Due to construction of the Agility‐MLC, the dose gradients in inplane and crossplane direction are different. For the steeper gradient in inplane direction [G‐T, Fig. 3(a)], the measured penumbra size was larger by 0.24 cm (Mx) and 0.27 cm (Oc) compared to Mc. This enlarged penumbra corresponds to almost twice the penumbra size using an ion chamber array, instead of the diode array Mc. In cross plane direction [A‐B, Fig. 3(b)] the penumbra is not as steep as in inplane direction. Also in crossplane direction, the penumbra size was smallest using Mc. Compared to Mc, the deviation was not larger than 0.15 and 0.17 cm for Mx and Oc respectively. Measurements in a large water phantom comparing an unshielded diode and an ion chamber (0.13 cm³) show a difference in penumbra size of 0.27 and 0.18 cm in inplane and crossplane direction, respectively.

### 3.B MLC parameters

#### 3.B.1 Picket fence test

**Figure 4:**
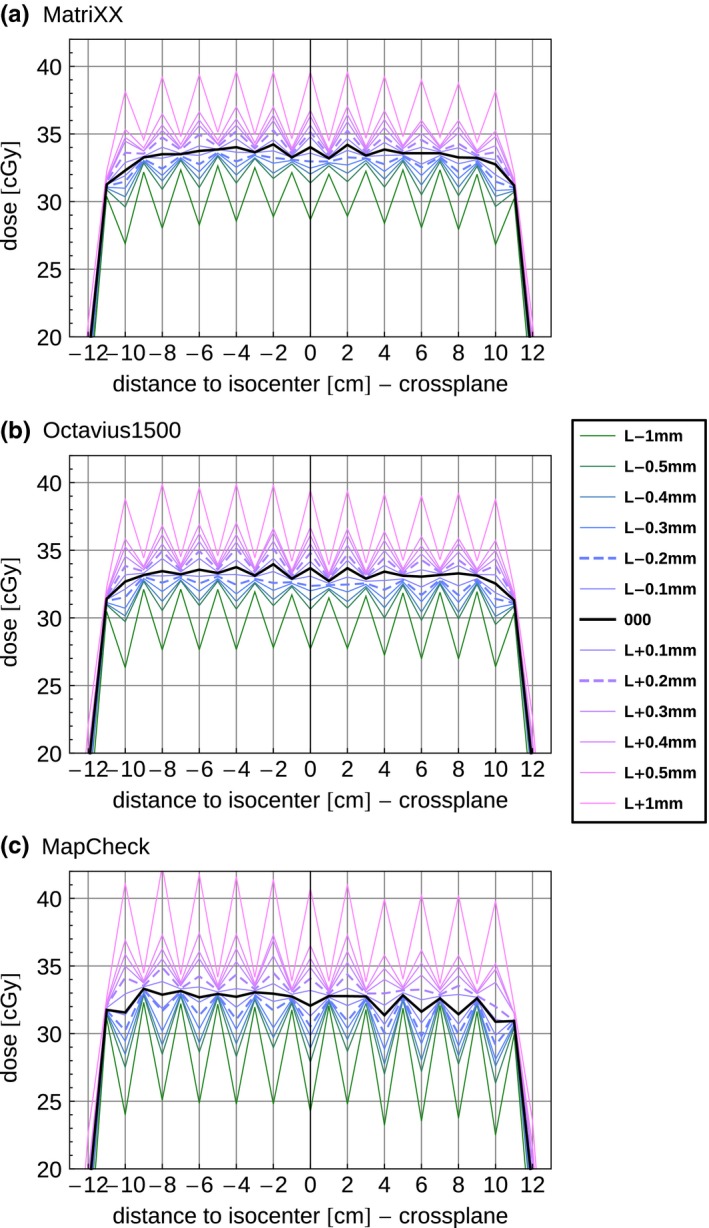
Static picket fence test with 12 × 2 cm wide segments for (a) Mx, (b) Oc and (c) Mc. Leaf manipulations: 0.1 → 1.0 mm: L+ refers to opening both leaf banks resulting in larger segments: L‐ refers to closing both leaf banks, resulting in smaller segments; 000 refers to the unmanipulated test. Mx was setup shifted in the short gantry mount (SSD = 76.2 cm) [Fig. 1(b)] and scaled to the isocenter; Oc and Mc were setup isocentric and shifted [Fig. 1(a)].

Altering the segment size, and thereby the overlap of the field abutment, would introduce dips or peaks. As Mc measured smaller dose gradients in general, the height of the peak (and depth of the dip) is more pronounced compared to the ion chamber arrays (Fig. 4). Therefore, Mc reliably detected leaf errors ≥0.1 mm in the isocenter plane compared to the unmanipulated measurements (000), while Mx and Oc detected leaf errors ≥0.2 mm (isocenter plane). Therefore, this test is potentially suitable to also detect these small errors in leaf miscalibration.

#### 3.B.2 Dynamic sweeping gap

**Figure 5:**
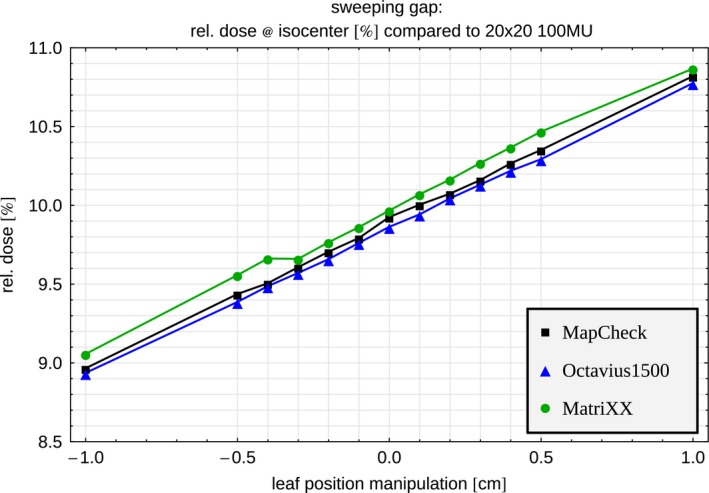
Sweeping gap test with a 2 cm wide gap swept over 20 cm across the field (100 MU) for the two innermost leaf pairs for Mx (green), and the leading leaf for Oc (blue) and Mc (black). Leaf manipulations: 0.1 → 1.0 mm (0.1 mm steps). The dose is calculated relative to the dose at the isocenter of a 20 × 20 cm² field.

The relative dose of the unmanipulated 2 cm wide sweeping gap with 100 MU compared to a 20 × 20 cm² field is 9.93%, 9.86%, and 9.97% for Mc, Oc, and Mx respectively (Fig. 5). Opening the leaves results in higher dose. All arrays show a linear dependence within 0.4% and the increase in dose is 10.1%/mm for Mc and Oc and 10.2%/mm for Mx when leaves are opened. This makes this test suitable to detect small leaf position errors on the same scale for all arrays.

#### 3.B.3 Chair test

**Figure 6:**
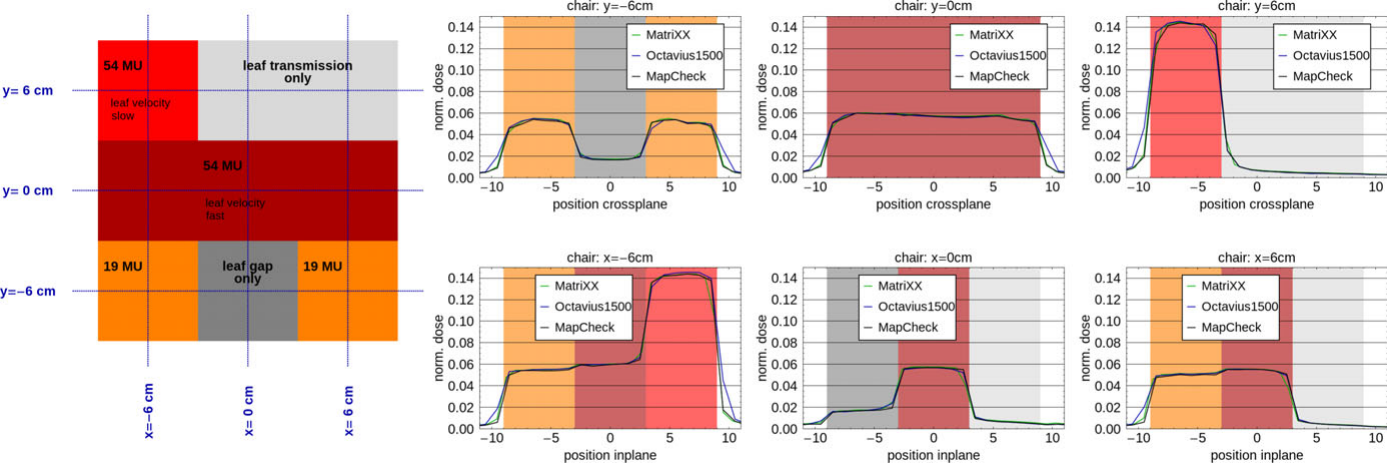
Adapted “chair” test: (a) overview of the different profile regions and the correcponding leaf properties, influencing the region, dashed lines: positions for profiles shown in b), and (b) profiles (dose normalized to 20 × 20 cm², 100 MU) along the in a) shown dashed lines for Mx (green), Oc (blue) and Mc (black).

Depending on how dose is delivered (leaf travel, transmission, or leaf gap only), the relative dose of the arrays differs, with Mc showing the lowest dose for all regions. In regions with leaf travel or leaf gap only, the deviation between the arrays is ≤1%. In the region with transmission only, the deviation is ≤2% (Fig. 6). This would result in slightly different values for leaf tip and leaf transmission values in the TPS, if different arrays are used for commissioning.

### 3.C Dynamic Interplay

#### 3.C.1 Interplay of dose rate and leaf movement

**Figure 7:**
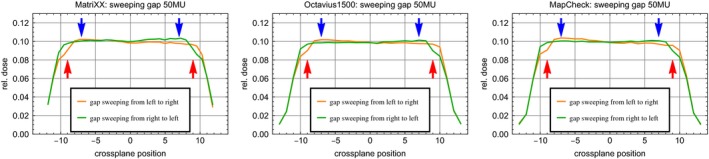
Sweeping gap test with a 2 cm wide gap swept over 20 cm across the field with 50MU from left to right (orange) and right to left (green) for Mx (left), Oc (middle) and Mc (right). The dose is calculated relative to the dose at the isocenter of a 20 × 20 cm² field. Red arrows indicate regions with up to 15% dose deviation in the starting region of the leaves; blue arrows indicate up to 5% overdose due to decreased profile homogeneity durung the linac ramp up at low dose rates.

For a sweeping gap (2 cm width, 20 cm travel distance) with different MU, the measured dose scales according to the difference of the MU within −1.9% for Mc and Oc and +1.2% for Mx, when the dose rate is around 200 MU/min (=20 MU delivered). For lower dose rates or doses, the deviation reaches −3.7%, −2.9%, and 5.6% for Mc, Oc, and Mx respectively. Inspecting the profiles of opposing leaf travel directions shows up to about 15% less dose in the starting region of the leaves, if the leaves are forced to accelerate with maximal leaf velocity. Furthermore, the decreased profile homogeneity of the starting beam and at low dose rates results in higher doses at the starting region of about 5% compared to a sweeping gap with reverse leaf movement direction (Fig. 7).

#### 3.C.2 Interplay of dose rate and gantry movement

Compared to the dose of an open field with the same MU, the dose of an open square field with gantry rotation (50° in each quadrant) changes in mean by 0.8%, 0.4%, and 0.4% for Mx, Oc, and Mc respectively. The (measured) homogeneity of the profiles was found to be more stable in quadrant 3 and 4 (gantry angle ≥90° and ≤270°), as well as for large MU and dose rate levels.

#### 3.C.3 Interplay of dose rate with gantry and leaf movement

**Figure 8:**
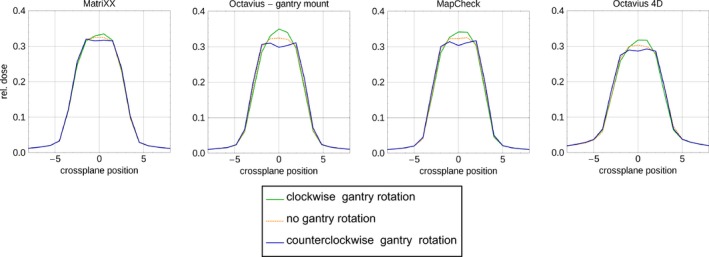
Sweeping gap test including gantry rotation (green) compared to the same sweeping gap without gantry rotation (orange) (field D, Table 1/C3) for Mx, Oc (in gantry mount), Mc and Oc4d (in rotational phantom); a) gantry rotation in clockwise direction; b) gantry rotation in counterclockwise direction.

The dose delivered by varying gantry speed, dose rate, and leaf speed (sweeping gap and simultaneous gantry rotation) was compared to the dose delivered by the same sweeping gap, but without gantry movement at gantry angle 0°. The two fields with full 360° gantry rotation (field B, D, Table 1/C3) show the largest difference, as the stability of the array in the gantry mount (and cylindrical phantom for Oc) and the leaves’ stability with respect to gravitation is more pronounced. Using the cylindrical phantom for Oc reduced the dose difference by about 35%. One of these full rotation fields (field D, Table 1/C3) showed higher dose than the corresponding field without gantry rotation: ΔD = 2.7%, 7.7%, 4.9%, and 4.8% for Mx, Oc, Mc, and Oc4D respectively (Fig. 8). Measuring the same field but with the opposite gantry rotation direction results in less dose compared to the nonrotation field: ΔD = −2.3, −6.6, −4.5, and −4.2% for Mx, Oc, Mc, and Oc4D respectively. This indicates that the gantry mount for Mx is more stable than the gantry mounts for Oc or Mc, and that the Oc array has a more stable position in the cylindrical phantom than in its gantry mount. The other six fields with gantry angles between 3 and 60° showed dose deviations not larger than 0.7%, 1.8%, 0.9%, and 1.3% for Mx, Oc, Mc, and Oc4D respectively.

## 4 DISCUSSION

During previous years, commercially available measurement devices for IMRT and VMAT QA have been tested for general performance and plan‐specific QA abilities^2^, ^3^, ^10^, ^24^, ^25^. Dose, dose rate and field size dependence as were described by these authors are supported by our data, even though the non‐linearity for very small doses in the linac ramp up was either not studied before or described to a different reference and also field sizes below 2 × 2 cm² we not studied. This study therefore contributes to more detailed understanding of array behavior in regions of small dose, dose rate, and field size, including effects of differences in profile homogeneity in static and dynamic delivery. Including three of the most often used 2D arrays (MatriXX^evolution^, Octavius^1500^, MapCHECK2) in one study offers the advantage of better comparability, which helps interpreting differences in gamma‐evaluation studies for plan‐individual QA, in general. Furthermore, decomposing the complexity of IMRT and VMAT into three categories (simple static QA, MLC parameters, dynamic interplay), we show the limitations of linac delivery and measurement devices with their implications for IMRT and VMAT planning, delivery, and different QA approaches. Our results support the ambition of introducing a linac based QA approach, which enables faster, simpler, and more meaningful QA procedures than is known from plan individual QA.

Different results for the arrays are mainly found due to the volume effect of ion chambers, which cause wider dose gradient regions and lower output factors for small field sizes (<2 cm × 2 cm). Therefore, also the static picket fence test does not detect the smallest (0.1 mm) introduced leaf miscalibration, using ion chamber arrays. The other tests showed slightly different results between the arrays, which may cause different plan QA results besides the volume effect. These results include the measurement of profile homogeneity, dose rate dependence, small dose measurements as well as different positional accuracy of the arrays in the dedicated mount for rotational measurements (gantry mount or cylindrical phantom). Therefore, either method, which compares the calculated dose distribution against the measured, will give different results for different arrays. Additionally, using one array, different plans having different characteristics with regard to e.g. the amount of small dose segments may also differ in the optimal parameters to set for the evaluation. Consequently, the found array characteristics have to be kept in mind during plan individual QA.

Searching for optimal the QA method, two main different ways are possible from the view of the authors: one way is perusing the plan individual QA; the other way is working towards linac based QA.

In general, plan‐individual QA focuses the challenge of finding the correct pass criteria. On the one hand, using the popular method of gamma‐evaluation and subsequent pass rate analysis,^26^ one can argue about the correct distance‐to‐agreement and dose criteria or pass rate, that has to be used.^2^, ^9^, ^10^, ^12^, ^13^, ^15^, ^18^ On the other hand, one can question this method in general,^27^, ^28^ as there might be a lack of correlation between the results of the gamma‐analysis and the clinical implication.^9^, ^11^, ^13^, ^14^, ^27^, ^29^, ^30^, ^31^, ^32^ Furthermore, the optimal criteria might depend on the measurement device,^11^, ^16^, ^17^, ^18^ which is supported by our results.

Pursuing plan individual QA, the found characteristics of the used array and linac have to be considered either in the comparison method or during the treatment planning process. Accounting for the characteristics of the measurement device and linac in the comparison method could imply increasing the tolerance in regions of steep dose gradients, if ion chamber arrays are used, and increasing the tolerance in regions of the dose distribution where the dose accumulates from large portions of small doses, small dose rates, and small field sizes. This tolerance may depend on the used array, the exact composition of dose distribution as well as the used linac. However, increasing tolerances will also mask important shortcomings of the plan and its delivery. Therefore, this procedure will not only be cumbersome but also misleading with regard to identify important delivery errors.

Instead of introducing location specific tolerances in plan QA, one could include the found characteristics of the measurement device and linac in the treatment planning system, for example by lookup tables. As this would imply unchanged characteristics of the linac during its life time, the QA procedure might get even stricter. Generating plans that respect shortcomings of the linac delivery as well as the shortcoming of used plan QA tool, e.g. by setting quite high constraints to the minimal dose per segment, may degrade the plan as the necessary degrees of freedom of IMRT and VMAT cannot be fully utilized.

Therefore, tight linac QA, which guarantees the TPS assumption within requested limits, may give enough confidence about the correct dose delivery of the plans. One part is finding the limiting factors that will degrade plan delivery and thereby change the dose distribution clinical significantly. According to the presented results, this would especially include the delivery conditions at linac ramp up and low dose rates, since larger deviations (e.g. from dose and dose rate linearity) were found at these conditions. Furthermore, dynamic techniques may not deliver dose as calculated, if leaves have to be driven at large speed and the dose rate has to change rapidly at the same time. Consequently, a second part toward linac QA consists of decomposing IMRT plans with respect to factors that may limit delivery quality and implement them in the treatment planning process and to predict its clinical influence for the specific plan. Eventually, this would lead to a plan specific constraint in the TPS, which monitors the potential amount of delivery outside of the given limitations. The quality of plan delivery will then depend on the range of essential linac parameters and the amount of dose delivery close to or outside the given limits. While the amount of dose delivered close to or outside of linac limits may depend on the specific requirements of the plan, the range of factors limiting delivery quality has to be kept small by meaningful tests, which are fast and easy to handle. The tests presented here will be suitable to not only detect delivery quality limiting parameters but also to quantify the resulting limits. Especially the dose linearity, dose rate linearity, profile stability with respect to dose, dose rate and gantry position as well as MLC positioning stability using the sweeping gap should be included into regular linac based QA and must be adapted to the individual measurement device and linac as well as to department specific requirements.

Implementing linac QA in the here suggested manner has the potential to fasten and facilitate IMRT and VMAT QA, which gives more meaningful results than widely used plan individual QA. The suggested decomposition of IMRT and VMAT into different complexity levels avoids cumbersome as well as time‐consuming plan individual QA measurements, which often need special according devices and comparison methods. The suggested procedure of implementing delivery shortcomings into the treatment planning process may avoid patient plans with bad delivery quality already at the planning stage.

The presented results also show the differences in several 2D arrays, which give rise to the assumption that the used 2D arrays are suitable for constancy test in the context of linac based QA, but might lack accuracy in initial linac commissioning. This is because the comparison of the results between different arrays may give different assumptions of the linac's condition and if actions need to be taken or not. Therefore, initial linac commissioning should be done using the known measurement devices, like single point detectors, which are well understood and known to have only small deviations in important linac characterization (e.g. small dose measurements).

The results of this study concerning the linac characteristics – especially during the ramp up – may be prone to a single misadjusted linac. Therefore, main results were verified on two matched linacs and showed the same results. Still the instabilities during ramp up might be limited to the construction of the specific type of linac used and should be verified for each single linac. Consequently, if limitations are found, these should be respected using either QA method (plan individual or linac based QA). The presented measurements could easily be translated to other measurement devices; the implementation procedure for a linac based QA approach would not differ.

## 5 CONCLUSION

Based on measurements with three different commercially available 2D arrays, we suggest linac based QA tests and how to derive threshold ranges for linac parameters, potentially influencing the delivery quality of IMRT and VMAT plans, which should be carefully considered during beam modeling and individual plan generation. These threshold ranges include restricting small MU per segment for step‐and‐shoot IMRT as well as fast traveling leaves at rapidly changing dose rates and small dose rates for dynamic IMRT techniques (dynamic sliding window and VMAT) due to nonlinearity of the beam homogeneity, especially during linac ramp up. Furthermore, the calibration point for the dose rate should not be at the maximal possible dose rate but around the most often used value of delivered IMRT and VMAT plans.

These parameters with their threshold ranges should be checked regularly together with the MLC positioning accuracy using a sweeping gap test as well as dynamic interplay tests that include various gantry rotation speeds and angles, various leaf speeds and various dose rates.

All used arrays are suitable for the suggested tests, even though small fields, steep dose gradients, low doses and beam profile homogeneity will be measured differently. Therefore, each array has its own limitations that need to be considered during plan individual QA as well as linac based QA. These limitations restrict the comparability of results measured with the different arrays with respect to plan individual QA as well as linac based QA, which makes the arrays potentially more suitable for constancy test, in general.

## CONFLICT OF INTEREST

The authors declare no conflict of interest.
